# Transient Adenosine Modulates Serotonin Release Indirectly in the Dorsal Raphe Nuclei

**DOI:** 10.1021/acschemneuro.3c00687

**Published:** 2024-02-09

**Authors:** Kailash Shrestha, B. Jill Venton

**Affiliations:** Department of Chemistry, University of Virginia, Charlottesville, Virginia 22901, United States

**Keywords:** neuromodulation, adenosine, serotonin, transient, FSCV, dorsal raphe nuclei (DRN), 5HT_1A_ antagonist

## Abstract

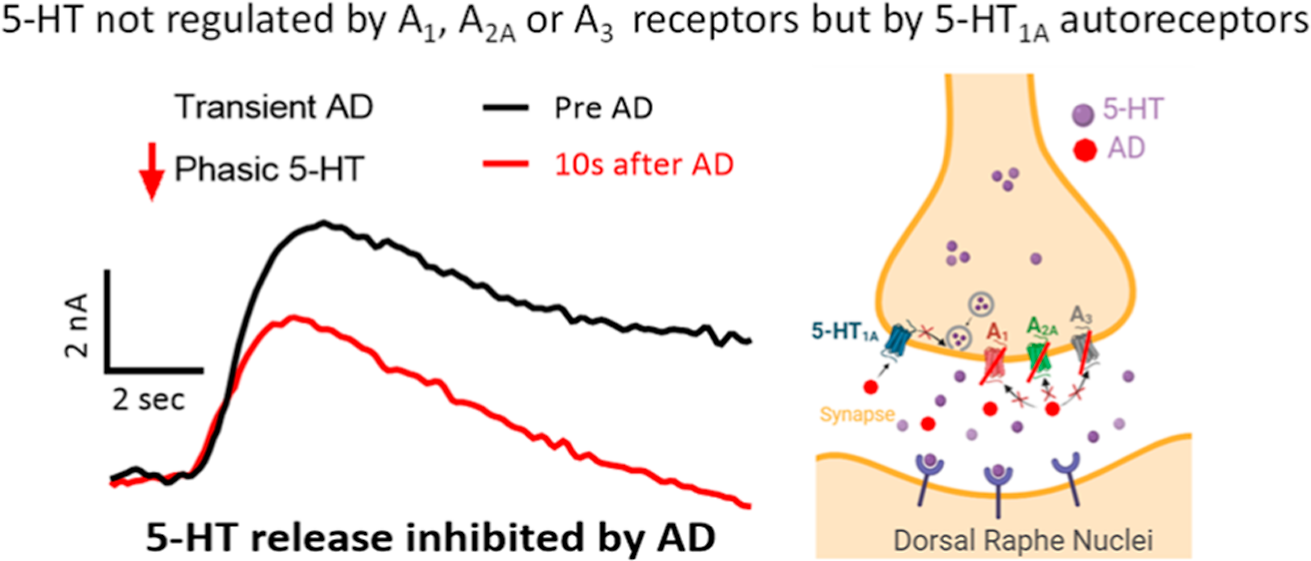

Rapid adenosine transiently regulates dopamine and glutamate via A_1_ receptors, but other neurotransmitters, such as serotonin, have not been studied. In this study, we examined the rapid modulatory effect of adenosine on serotonin release in the dorsal raphe nuclei (DRN) of mouse brain slices by using fast-scan cyclic voltammetry. To mimic adenosine release during damage, a rapid microinjection of adenosine at 50 pmol was applied before electrical stimulation of serotonin release. Transient adenosine significantly reduced electrically evoked serotonin release in the first 20 s after application, but serotonin release recovered to baseline as adenosine was cleared from the slice. The continuous perfusion of adenosine did not change the evoked serotonin release. Surprisingly, the modulatory effects of adenosine were not regulated by A_1_ receptors as adenosine still inhibited serotonin release in A_1_KO mice and also after perfusion of an A_1_ antagonist (8-cyclopentyl-1,3-dipropyl xanthine). The inhibition was also not regulated by A_3_ receptors as perfusion of the A_3_ antagonist (MRS 1220) in A_1_KO brain slices did not eliminate the inhibitory effects of transient adenosine. In addition, adenosine also inhibited serotonin release in A_2A_KO mice, showing that A_2A_ did not modulate serotonin. However, perfusion of a selective 5HT_1A_ autoreceptor antagonist drug [(S)-WAY 100135 dihydrochloride] abolished the inhibitory effect of transient adenosine on serotonin release. Thus, the transient neuromodulatory effect of adenosine on DRN serotonin release is regulated by serotonin autoreceptors and not by adenosine receptors. Rapid, transient adenosine modulation of neurotransmitters such as serotonin may have important implications for diseases such as depression and brain injury.

## Introduction

Adenosine is a widely distributed neuromodulator in the central nervous system that modulates neurotransmission and plays a neuroprotective role via adenosinergic, G protein-coupled receptors.^1,2^ Four subtypes of membrane-bound adenosine receptors exhibit inhibitory (A_1_ and A_3_) and excitatory effects (A_2A_ and A_2B_) by altering adenylyl cyclase activity at the synaptic terminal.^1,3−6^ During pathological conditions, extracellular adenosine accumulates at different rates. Slower accumulation during damage or stroke can last for hours and prevent further tissue damage by reducing excitatory neuronal firing at the synapse.^6−9^ However, there are rapid modes of adenosine release which last for just a few seconds.^10,11^ Dunwiddie et al. discovered transient adenosine signaling that ranged from milliseconds to seconds, with rapid neuromodulatory effects in the hippocampus.^12^ Similarly, using fast-scan cyclic voltammetry (FSCV), our lab characterized spontaneous transient adenosine, lasting 2–3 s, that modulates neurotransmitter release and is released in response to ischemia.^9,11,13,14^ Mechanical perturbation of tissue also generates transient adenosine, but mechanically stimulated adenosine is higher in concentration and lasts around 20–30 s with a broader effective area.^4,9^ This mode of rapid adenosine release may serve a neuroprotective role during physical injuries and trauma. Adenosine, a well-known retaliatory metabolite, modulates the synaptic release of other neurotransmitters such as dopamine (DA), glutamate, and GABA through its presynaptic and postsynaptic receptors.^1,11,15^ Basal changes in adenosine have been reported to modulate serotonin release in the hippocampus, but there have been no investigations of the effect of rapid changes in adenosine to modulate serotonin release in the dorsal raphe nuclei (DRN), which is responsible for cognition, sleep, and locomotion.^16−18^

The DRN and medial raphe nuclei are the largest serotonergic clusters in the brain.^19,20^ A wide distribution of serotonin neurons and an abundance of serotonin receptors contribute to serotonin’s broad modulatory roles and diverse physiological functions, such as regulating cognition, the sleep–wake cycle, aggression, locomotion, and mood.^21^ These excitatory serotonin neurons are closely regulated by the abundant somatodendritic 5-HT_1A_ autoreceptor, a G protein-coupled receptor.^18,20,21^ In addition, inhibitory GABAergic neurons, glutamatergic neurons, and cholinergic neurons alter neuronal firing through changes in K^+^ and Ca^2+^ channel permeability.^16,18,22^ However, the direct modulatory role of adenosine on serotonin release has not been well understood, particularly in the DRN.

Adenosine has a modulatory effect on neurotransmitters, and direct measurements of these neurotransmitters help elucidate these effects. For example, Ross et al. demonstrated the inhibitory action of transient adenosine on dopamine by 50% via A_1_ receptors in the caudate putamen.^11^ Similarly, using superfusion techniques, Feuerstein et al. found that both theophylline and 8-phenyl theophylline, A_1_ receptor antagonists, abolished the inhibitory effect of endogenous adenosine via A_1_ receptors on stimulated serotonin release in the rabbit hippocampus slice.^23,24^ Thus, A_1_ receptors may regulate serotonin release.^25,26^ Furthermore, adenosine deaminase, an enzyme responsible for adenosine metabolism, is present in the DRN, and thus, adenosine may regulate serotonin in the DRN.^27^ In the previous study by Feuerstein et al., the modulatory effect of adenosine was studied in a slower time scale using the superfusion technique, in which isotopic serotonin samples were collected every 60 min and analyzed.^28^ Although 5-HT_1A_ autoreceptors and GABAergic receptors tightly regulate serotonin concentrations, there are no studies that address the modulation of transient adenosine on serotonin release in the DRN.^29,30^

The goal of this study was to test the modulatory effect of transient adenosine on serotonin release in DRN in real-time. Rapid adenosine and serotonin release were measured using FSCV, a highly sensitive real-time detection technique with subsecond temporal resolution^31,32^ after puffing adenosine, which lasted 25 s, mimicking mechanosensitive release. Transient adenosine inhibited serotonin by 40% in WT in the first 20 s of adenosine application, but this same effect was not observed during adenosine perfusion. A_1_KO mice and an A_1_ antagonist were used to investigate the role of inhibitory A_1_ adenosine receptors, but there was no change in adenosine modulation of serotonin in either model. Similarly, inhibition of serotonin was not abolished in A_2A_KO mice or using a selective A_3_ antagonist in A_1_KO mice. However, the adenosine modulation of serotonin was eliminated during the perfusion of a selective 5HT_1A_ antagonist. Thus, adenosine modulates serotonin dynamics in the DRN, not through adenosine receptors but indirectly through 5HT_1A_ autoreceptors.

## Results and Discussion

This study tests the hypothesis that rapid adenosine transiently downregulates DRN serotonergic release. To assess how adenosine modulates serotonin release, we measured stimulated serotonin in a DRN brain slice by implanting a cylinder carbon–fiber microelectrode (CFME) and using FSCV. Twenty-five pmol of adenosine was locally microinjected via picospritzer at different time intervals (10, 20, 40, and 80 s) before serotonin stimulation. Exogenously puffed adenosine lasts ∼25 s, which is similar to mechanically stimulated adenosine release.^11^

### Rapid Changes in Local Adenosine Modulate DRN Serotonergic Neurons in WT Mice

**Figure 1:**
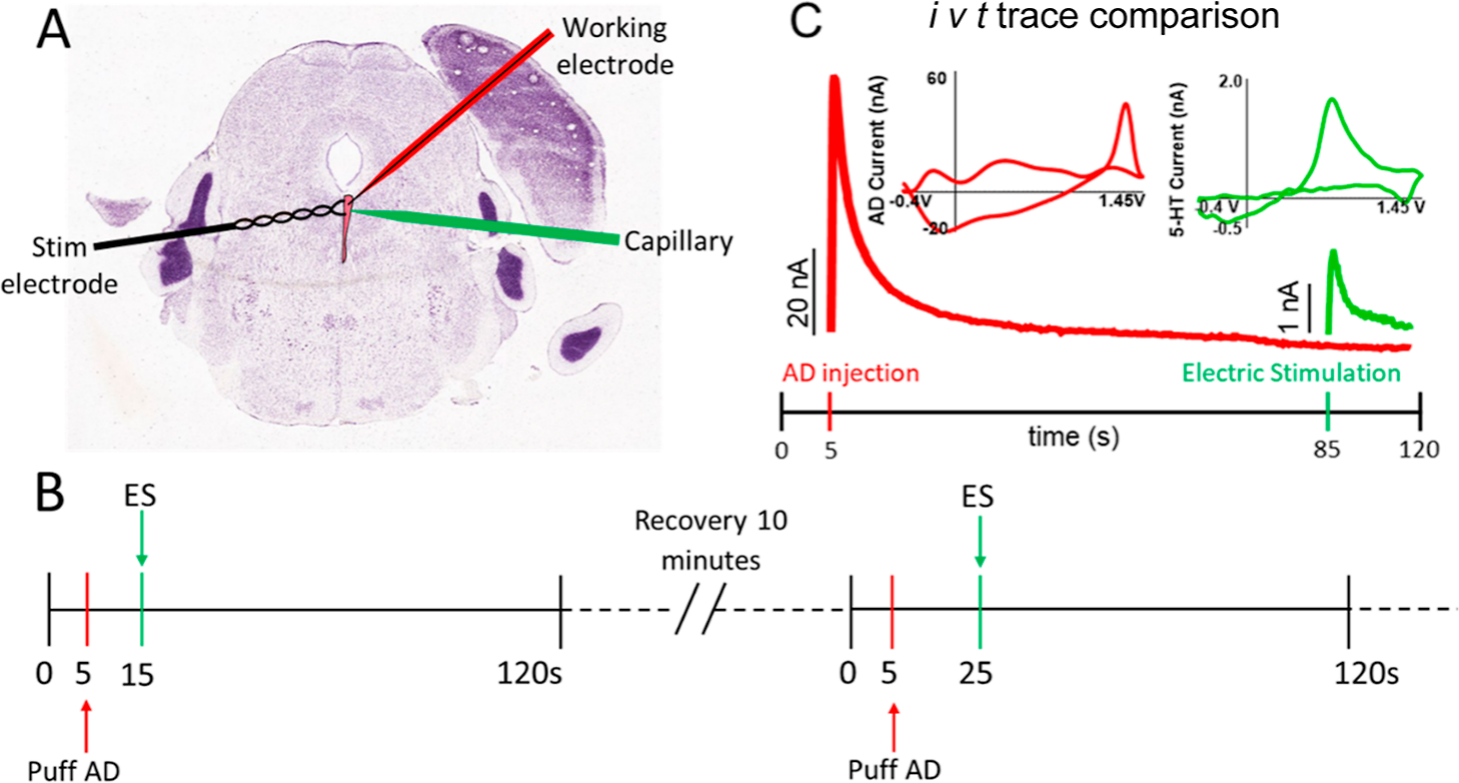
Experimental design. (A) Experimental setup showing CFME, stimulating electrode, and glass capillary for adenosine injection using a picospritzer in the DRN. (B) An experimental timeline shows adenosine injection at 5 s (red arrow), followed by stimulation (green arrow) after 10 s. The interval between adenosine application and stimulation is increased, i.e., 10, 20, 40, and 80 s, with the following scans, and finally, recovery scan was performed without adenosine (not shown in the timeline). There was 10 min between experiments. (C) Representative example: 1 mM adenosine (50 pmol) is administered via pico-spritzer (10 psi, 10 ms, 100 nL) 80 s prior to serotonin stimulation, as shown with the i vs t curve for adenosine (red trace) and serotonin (green trace). The inset contains an adenosine cyclic voltammogram (red) with two oxidation peaks at +1.4 V (primary) and +1.0 V (secondary) and a serotonin cyclic voltammogram with an oxidization potential at 0.7 V (green), indicating that both analytes can be detected using an adenosine waveform.

First, we measured the effect of transient adenosine on the stimulated serotonin in WT mice. Figure 1A shows the experimental setup for this study. The working electrode (CFME) and the capillary with adenosine were inserted into the tissue at about 50 μm apart. Although many other waveforms such as the Jackson waveform and the extended serotonin waveform have been developed to more selectively monitor serotonin, we used the adenosine waveform (−0.4 V, 1.45 V; scan rate: 400 V/s) to monitor both adenosine and serotonin in the same scan.^33−35^ The main interferent for serotonin with that waveform would be dopamine, but there are no dopaminergic neurons in the DRN, so dopamine is not an interferent for serotonin detection. Figure 1B summarizes the experimental design, where serotonin was electrically stimulated at different time intervals (10, 20, 40, and 80 s) after adenosine injection. Stimulations were repeated every 10 min to allow recovery of release, and this time interval produces the same release for all stimulations.^36^Figure 1C shows stimulated serotonin 80 s after adenosine application. Adenosine is cleared from DRN around 20 s after injection, as seen in the *i-t* curve (top inset, red trace). This *i-t* curve mimics mechanically stimulated adenosine, which is quickly released and elevated for about 20–30 s before it is fully cleared from the extracellular space.^9^ FSCV is a background-subtracted method that monitors the rapid change in electroactive analyte concentration. The background subtracted CV, the inset, is a signature CV for serotonin that confirms the identity of the release of serotonin. Supporting Information Figure 1 shows an example color plot of electrically evoked serotonin 20 s after adenosine injection, at two different color contrasts to show the adenosine (which has a higher concentration and produces larger oxidation currents) and serotonin. We measured serotonin levels from the *i* vs *t* curve at the oxidation potential for serotonin.

**Figure 2:**
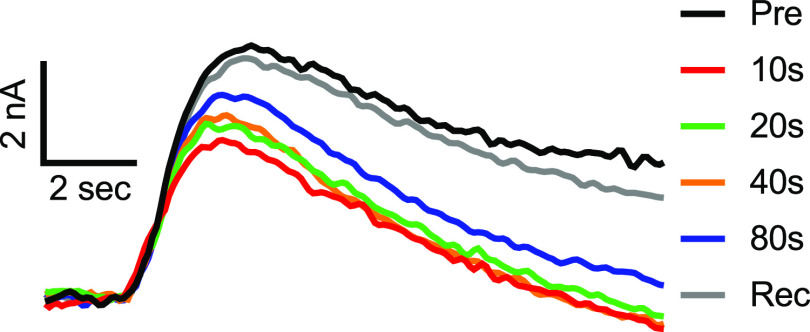
Serotonin (5-HT) release i-t curve with adenosine at different time intervals (10, 20, 40, and 80 s) and without adenosine. ES (pre-AD) and Rec are after the AD trials. The *y*-axis is the serotonin current (nA) detected after electrically evoked serotonergic release in the DRN. The *x*-axis is time (s). AD: adenosine; ES: electric stim.

Figure 2A shows overlapped serotonin release traces before and after adenosine was puffed at different time intervals. Adenosine suppresses electrically evoked serotonin release in the DRN, particularly when the time interval between the adenosine application and stimulation is small. As the time interval between adenosine injection and serotonin stimulation increases, serotonin release gradually increases back to baseline. The recovery curve (gray trace) shows that serotonin release returns to normal when no adenosine is applied to the slice at the end of the experiment, showing that the tissue is viable. Thus, when adenosine is present, stimulated serotonin is inhibited.

**Figure 3:**
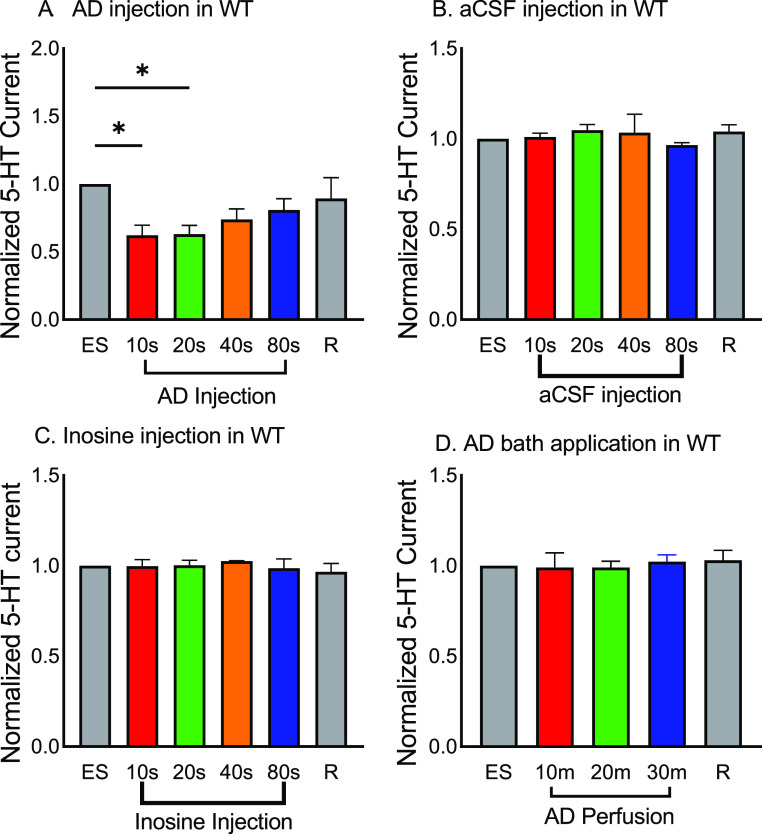
Neuromodulatory effect of adenosine on stimulated serotonin. (A) Effect of transient adenosine. 50 pmol of adenosine was exogenously puffed after an electrical stimulation at different time intervals (10, 20, 40, and 80 s) in the DRN of a WT brain slice. Each bar is serotonin current normalized to the first stimulation without adenosine [black: initial ES; red: adenosine applied 10 s before; green: 20 s; orange: 40 s; blue: 80 s; gray: recovery (no adenosine at the end)]. There is a significant effect of the time interval for adenosine on serotonin release (one-way ANOVA, *n* = 7; *p* = 0.0291). (B) Transient aCSF control. 0.1 M HClO_4_ was diluted in aCSF as adenosine stock was and then applied instead of adenosine, and no significant effect was observed on serotonin (one-way ANOVA, *n* = 4; *p* = 0.8221). (C) Inosine (10 μM) injection in the DRN at different time intervals to test the effect of an adenosine metabolite. There was no difference in serotonin release with or without inosine injection (one-way ANOVA, *n* = 3, *p* = 0.8888). (D) Bath application of adenosine (10 μM) for 30 min. Serotonin was stimulated every 10 min and normalized to the initial stimulated serotonin. Serotonin release did not differ with adenosine perfusion (one-way ANOVA, *n* = 3, *p* = 0.9621). Error bars are the SEM. ES: electric stim.

Figure 3A compares the averaged data for serotonin stimulations at various time intervals after adenosine application. The *y*-axis is current normalized to the initial, preadenosine-stimulated serotonin and the *x*-axis is the time interval between the adenosine administration and electrical stimulation. The gray bar is a final recovery stimulation without any adenosine, showing that the tissue is still viable and stimulated serotonin release has not changed over time. Adenosine decreased for the 10 and 20 s intervals and then gradually returned to its initial serotonin level with 40 and 80 s and recovery (Figure 3A). There is a significant main effect of the time interval of adenosine on serotonin release (one-way ANOVA; *n* = 7; *p* = 0.0291), and post-tests show that transient adenosine significantly reduced stimulated serotonin for the 10 and 20 s adenosine groups (Dunnett’s post-test: 10 s: red, *p* = 0.0195; 20 s: green, *p* = 0.0231). The 40 s, 80 s, and recovery groups are not significantly different from the initial stimulation (one-way ANOVA; *n* = 7; 40 s: orange, *p* = 0.1605; 80 s: blue, *p* = 0.4138; recovery: gray, *p* = 0.8638). Serotonin was suppressed by 40% for the 10 and 20 s groups, showing that transient adenosine decreased release by almost half. The adenosine i vs t trace (Figure 1C) reveals that adenosine is elevated in the tissue for about 20 s, so these data show that adenosine modulates serotonin only when it is transiently elevated. Thus, adenosine transiently modulates DRN serotonergic release when the level of adenosine is elevated.

As a control, we replaced the adenosine injection with an artificial cerebral spinal fluid (aCSF) buffer injection and recorded stimulated serotonin (Figure 3B). aCSF was injected at the same time intervals as in the adenosine experiment, and there was no significant effect of aCSF injection on the serotonin release (one-way ANOVA, *n* = 4; *p* = 0.8221). Finally, to make sure that the effect was due to adenosine and not its common downstream metabolite, we injected inosine, the major metabolite of adenosine, which is formed from adenosine deaminase breakdown. There is no significant effect of puffing on inosine at the various time intervals before stimulating serotonin (Figure 3C, one-way ANOVA, *n* = 3, *p* = 0.8888). This further confirms that adenosine is causing the neuromodulatory effects on serotonin release, and the decrease is not just an effect of puffing on a reagent or a metabolite.

After testing the effects of rapid adenosine neuromodulation, we tested the effects of bath application of adenosine, simulating an increase of basal adenosine levels. Figure 3D shows perfusion of 10 μM adenosine for 30 min, while stimulating serotonin every 10 min, and a recovery stimulation after perfusion was switched to normal aCSF. The bath perfusion did not suppress stimulated serotonin at 10, 20, or 30 min. Stimulated serotonin levels were not significantly different between pre- and postadenosine perfusion in the DRN region (one-way ANOVA; *n* = 4, *p* = 0.9621). Thus, these data confirm that the inhibition of serotonin release is mediated by transient adenosine but not with changes in basal adenosine levels.

Adenosine is a ubiquitous neuromodulator, predominantly known for its inhibitory function via G_i/o_ proteins on neurotransmitters such as dopamine, GABA, and glutamate.^7,23,24,37^ Here, we examined the extent to which adenosine regulates phasic serotonin release by mimicking mechanosensitive adenosine release, which elevates adenosine for about 20–30 s in the extracellular space.^9^ The main finding is that adenosine inhibits serotonin release only when it is transiently elevated. After adenosine is cleared, in approximately 20 s, stimulated serotonin release is not significantly inhibited. The maximum inhibition is about 40%, which is similar to the transient inhibition of dopamine by adenosine in the caudate putamen.^11^ Similarly, dopamine was inhibited only when adenosine was transiently elevated and not after adenosine had been cleared. Thus, there is a significant but partial depression of serotonin release in the presence of transient adenosine.

Early studies examined the effects of adenosine to regulate serotonin release and found that A_1_ receptors mediate adenosine inhibition of evoked serotonin in the hippocampus but not the caudate putamen.^23,24^ However, those studies did not examine transient adenosine or the modulation of adenosine in DRN serotonergic neurons. Here, we found that mimicking increases in basal levels of adenosine by perfusing adenosine did not have any inhibitory effect on phasic serotonin release. Thus, adenosine acts transiently to rapidly activate receptors and downregulate serotonin release, but not through the constant action of basal receptor occupancy. The main finding here is that adenosine inhibits serotonin release in the DRN only while it is transiently elevated. This rapid effect could be very important in temporarily suppressing neurotransmission during tissue damage or other trauma conditions.

### Transient Adenosine Suppression of Serotonin Release Is Not Regulated by A_1_ Receptors

Adenosine typically exhibits an inhibitory role predominantly via A_1_ receptors, which are located presynaptically.^7,27,38^ A_1_ receptors regulated the transient modulation by adenosine of dopamine,^11^ and here, we hypothesized A_1_ receptors would have the same role for serotonin. To examine the inhibitory effect of adenosine via A_1_ receptors, adenosine modulation of serotonin release was monitored in genetically modified A_1_KO mice and in WT mice slices perfused with 8-cyclopentyl-1,3-dipropyl xanthine (DPCPX), an A_1_ inhibitor.

**Figure 4:**
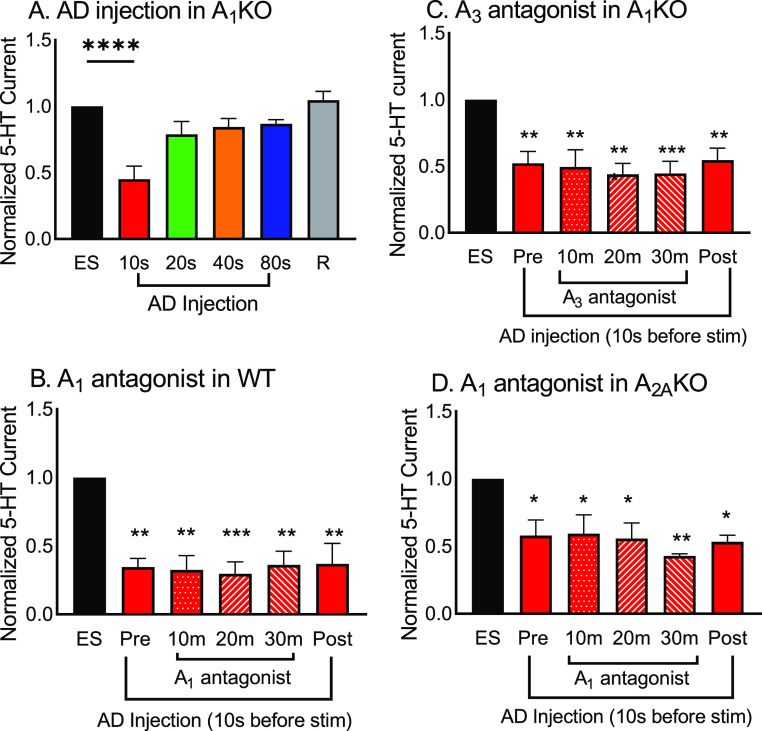
Effect of adenosine receptors to regulate the effects of transient adenosine on serotonin release. (A) AD injection in A_1_KO mice. The time interval was varied for adenosine application before serotonin stimulation, and serotonin is still suppressed by adenosine with the 10 s interval (one-way ANOVA, *n* = 7; ES; 10s: red, *p* < 0.0001). (B) A_1_ antagonist (10 μM DPCPX) perfused for 30 min in WT mice. Serotonin was stimulated 10 s after adenosine application, and DPCPX did not stop the inhibition of adenosine (one-way ANOVA, *n* = 3, *p* = 0.0016). (C) Effect of A_3_ antagonist (5 μM of MRS 1220) on inhibition of adenosine on serotonin release in A_1_KO mice. The drug had no effect as adenosine still inhibited serotonin (one-way ANOVA, *n* = 5; *p* = 0.0014). (D) A_1_ antagonist (10 μM DPCPX) perfused in an A_2A_KO slice with adenosine applied 10 s before serotonin stimulation. The A_1_ antagonist did not change the inhibitory effect of transient (one-way ANOVA, *n* = 3, *p* = 0.0127). The error bar represents the SEM. For B, C, and D, asterisks mark significant differences from predrug ES. *p < 0.05, **p < 0.01, ***p < 0.001 (AD: adenosine; ES: electric stim pre-AD).

Figure 4A shows serotonin release after adenosine application in A_1_KO mice, with the same protocol used for WT mice (10, 20, 40, and 80 s intervals, followed by recovery without adenosine). There was a significant main effect of adenosine on serotonin release in A_1_KO mice (one-way ANOVA, *n* = 7, *p* < 0.0001), and stimulated serotonin release was significantly inhibited by transient adenosine in the first 10 s of application (Dunnett’s post-test, *n* = 7, *p* < 0.0001). The decrease of serotonin to 45% of the initially stimulated serotonin was similar to that of WT. However, serotonin suppression was not significantly different from the initial stimulation for the 20 s time interval in A_1_KO mice, unlike that of WT (Dunnett’s post-test; 20s: green, *p* = 0.1373, *n* = 7). This slight increase in serotonin release after 20 s of adenosine application in A_1_ knocked-out mice could be due to compensatory overexpression of another inhibitory receptor, such as A_3_ adenosine receptors or GABA receptors, that have a different time frame of interaction.^39^ Similar to WT, stimulated serotonin release gradually recovers to baseline with no significant differences from the initial stimulation for transient adenosine with 40 s, 80 s, or recovery. The large inhibition that occurs at 10 s indicates that A_1_ receptors are not primarily responsible for the inhibition of serotonin in the DRN.

To further ensure A_1_ receptors have no role in this inhibition by transient adenosine, we perfused the A_1_ antagonist drug DPCPX (10 μM) in WT mice for 30 min and stimulated serotonin release every 10 min with adenosine applied 10 s before (Figure 4B). All serotonin currents were normalized to the initial stimulation with no adenosine. There was a significant effect of adenosine on stimulated serotonin release (one-way ANOVA, *n* = 3, *p* = 0.0016), and serotonin release was inhibited by adenosine even during perfusion of DPCPX. Thus, A_1_ receptors are not responsible for mediating the inhibitory effect of transient adenosine on serotonin.

The vast majority of studies demonstrating inhibitory effects of adenosine have shown A_1_ receptors that mediate these effects; thus, our results are surprising.^4,7,24^ For example, the inhibition of serotonin release in the hippocampus by adenosine was blocked by an A_1_ antagonist, 8-phenyltheophylline, indicating A_1_ receptors mediate the inhibitory effect.^23^ Adenosine modulation of dopamine release was also mediated by A_1_ receptors in the caudate putamen and hippocampus.^5,11,15^ In the DRN, A_1_ receptors are expressed and adenosine deaminase is present, an enzyme responsible for adenosine metabolism, suggesting adenosine plays a neuromodulatory role there.^25−27^ In A_1_KO mice, there is still a strong inhibitory effect of adenosine applied 10 s before serotonin stimulation, indicating that A_1_ receptors are not primarily mediating the inhibition. There were some slight variations in the 20 s data, which are not significant in A_1_KO mice but were in the WT mice, but the overall inhibition after 10 s is just as strong as the WT mice. Pharmacological experiments with DPCPX, an A_1_ antagonist, in WT mice also verify that A_1_ receptors do not mediate the inhibition of serotonin release. These data are surprising because of the evidence of A_1_ inhibition in other studies, but here, A_1_ receptor blockade is not sufficient to remove the inhibition of serotonin by adenosine. Thus, A_1_ receptors are not the primary receptors mediating the inhibitory action of adenosine on DRN serotonergic release.^11,24^

### Inhibitory Effect of Adenosine on Serotonin Release Is Not Mediated by A_3_ or A_2A_ Receptors

Since A_1_ receptors are not primarily responsible for the suppression of DRN serotonin release, we tested the effects of A_3_ receptors, which are also inhibitory and might be upregulated in A_1_KO mice.^39^ A_3_ receptors are also inhibitory with lower affinity, although they are more sparsely located than A_1_ receptors.^1,4,7,37^ An A_3_ antagonist (MRS 1220, 5 μM) was perfused in the A_1_ knockout slice. The A_1_KO was used to eliminate both of the possible inhibitory receptors at once when the antagonist was perfused. We perfused MRS 1220 for 30 min while stimulating serotonin 10 s after adenosine application every 10 min (Figure 4C), before washing out the drug for a recovery stimulation with adenosine application. There is still a significant effect of adenosine on serotonin release (one-way ANOVA, *n* = 3, *p* = 0.0014) and no differences with the A_3_ receptor antagonist. Thus, adenosine still clearly inhibited serotonin release, even in an A_1_KO slice in the presence of an A_3_ antagonist. These results show that neither of the two major inhibitory receptors, A_1_ or A_3_, directly mediates the inhibition of serotonin by transient adenosine.

A_2A_ receptors are the main excitatory receptors for adenosine, and they are the second most abundantly distributed receptors in the CNS.^2,4,5,7^ The activation of these excitatory receptors, coupled with G_s/olf_, activates presynaptic receptors to stimulate neurotransmitter release. However, these excitatory receptors can form heteromers with inhibitory A_1_ adenosine receptors at the same terminal, and they modulate glutamate neurotransmitter release.^40^ Thus, although A_2A_ receptors are excitatory, we tested their effects. We used A_2A_ knockout mice along with DPCPX to block both A_1_ and A_2A_ receptors to test if heteromers were responsible for the inhibition. A similar experimental setup was used as with the A_3_ antagonist data. There was a main effect of adenosine in regulating serotonin release (Figure 4D, one-way ANOVA, *n* = 3, *p* = 0.0127). Serotonin release was significantly suppressed by adenosine in the A_2A_ knockout slice when adenosine was injected 10 s before electric stimulation (one-way ANOVA, *n* = 3, *p* = 0.0257). When 10 μM of DPCPX was perfused, stimulated serotonin after adenosine was still significantly inhibited compared to the initial stimulation (one-way ANOVA, *n* = 3, *p* = 0.0311). Thus, in A_2A_KO mice, serotonin release is still inhibited by transient adenosine applied 10 s before, and when perfusing 10 μM DPCPX in the A_2A_ knockout mice, adenosine still inhibits serotonin release. These data show that A_2A_ receptors are not responsible for the inhibitory effects of adenosine and that heteromers are also likely not the source of adenosine modulation. Thus, surprisingly, no adenosine receptors were identified that mediate the inhibitory effect of transient adenosine on serotonin release.

### Selective 5HT_1A_ Antagonist Blocks the Effects of Adenosine on Serotonin Release

Our data showed that transient adenosine still inhibits electrically evoked serotonin release while blocking A_1_, A_2A_, or A3 receptors. Since no adenosine receptors were responsible for the inhibition by transient adenosine, we tested if 5HT_1A_ autoreceptors, most abundantly found in the DRN, regulate the inhibition of serotonin by adenosine.^21^ These presynaptic 5HT_1A_ autoreceptors are densely packed at DRN and regulate serotonergic release through a negative feedback loop.^18,21^

**Figure 5:**
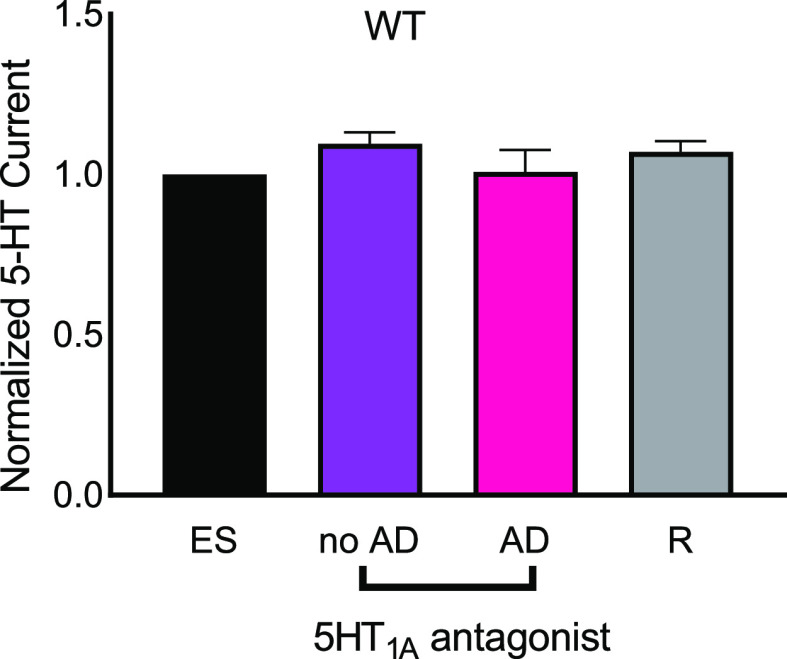
Adenosine modulation of serotonin release in the presence of selective 5HT_1A_ antagonist perfusion [(S)-WAY 100135 dihydrochloride, 5 μM]. Bars are normalized to the first serotonin stimulation (black). In the presence of WAY 100135, serotonin was stimulated without and then with adenosine applied 10 s before. The recovery is stimulated by serotonin with normal aCSF. There is no significant difference in stimulated serotonin (one-way ANOVA, *n* = 3; *p* = 0.3623).

The selective 5HT_1A_ antagonist (S)-WAY 100135 dihydrochloride (5 μM, 5HT_1A_ IC_50_ = 34 nM) was perfused over a WT slice for 30 min.^41^Figure 5 shows the data. An electrical stimulation was performed without drug, then WAY 100135, a 5HT_1A_ antagonist, was perfused, and an electrical stimulation was performed with no adenosine. The 5HT_1A_ antagonist did not alter electrically stimulated serotonin levels, similar to previous microdialysis studies that found that 5-HT_1A_ antagonists alone do not increase serotonin.^42^ Similar effects have been observed in previous studies using electrophysiology, where the 5-HT_1A_ agonist 8-OH-DPAT [7-(dipropylamino)-5,6,7,8-tetrahydronapthalen-1-ol] also did not change serotonin levels.^41,43^ Then, in the presence of a 5HT_1A_ antagonist, an electrical stimulation was performed with adenosine applied 10 s before, and finally, a recovery stimulation with no adenosine and no drug was performed. There was no effect of adenosine on stimulated serotonin release in the presence of a 5HT_1A_ antagonist (one-way ANOVA, *n* = 3; *p* = 0.3623). Predrug-stimulated serotonin release was similar to stimulated serotonin release in the presence of the 5-HT_1A_ antagonist and with adenosine puffed 10 s before serotonin stimulation. With perfusion of the 5HT_1A_ antagonist drug, the inhibitory effect of transient adenosine was eliminated, indicating that 5HT_1A_ autoreceptors tightly regulate DRN serotonergic release and at least indirectly mediate the inhibitory effect of adenosine. Future studies could also use different 5-HT_1A_ autoreceptor drugs to confirm the specificity of the drug, but these data suggest that serotonin autoreceptors are regulating the effects of adenosine to modulate serotonin.

Serotonin receptors tightly regulate their own local vesicular release through inhibitory 5-HT_1A_ autoreceptors.^44^ These inhibitory somatodendritic autoreceptors are associated with K^+^ channels via G protein-coupled inwardly rectifying K^+^ (GiRK) channels, which closely regulate basal serotonin levels via a negative feedback loop.^16,18^ Activation of Gα_i/o_-coupled serotonin autoreceptors upregulates K^+^ conductance, hyperpolarizes neuronal membranes, and generates fewer action potentials that suppress neurotransmitter release in the extracellular space.^45^ A_1_ adenosine receptors, GABA receptors, 5-HT_1A_ receptors, and D_1_/D_2_ dopamine receptors are some of the common receptors coupled with GiRK channel activation via GPCRs in the brain.^46^ A potent 5HT_1A_ antagonist [(S)-WAY 100135 dihydrochloride, 5 μM], which blocks both pre- and postsynaptic 5HT_1A_ autoreceptors, was perfused to examine if autoreceptors regulated the inhibition of serotonin release by adenosine.^41^ Stimulated serotonin levels do not change in the presence of the 5HT_1A_ antagonist, similar to previous studies.^42^ Interestingly, when transient adenosine was applied 10 s before serotonin stimulation in the presence of a 5HT_1A_ antagonist, there was no suppression of the serotonin release. Thus, 5HT_1A_ inhibition blocked the inhibition of serotonin by transient adenosine.

The exact mechanism of how adenosine interacts with the serotonin autoreceptors is unknown. It is doubtful that adenosine acts directly at the serotonin autoreceptors as no affinity for adenosine is known at serotonin receptors. Activation of 5-HT_1A_ leads to decreased cAMP concentrations; thus, adenosine may activate this pathway as well by inhibiting adenylate cyclase, producing cAMP.^47^ There is a possibility of an indirect inhibition pathway or cross-coupling with other channels such as GiRK mediated by G-βγ subunits and Ca^2+^ channels that adenosine activates that feedback on serotonin levels or serotonin autoreceptors.^48^ Adenosine increases GiRK channel conductance via A_1_ receptors, which further depolarizes neuronal action potential and suppresses stimulated release in the hippocampus.^46,49^ Similarly, changes in GiRK channel permeability are also associated with inhibitory GABA_B_ and autoinhibitory 5HT_1A_ receptors, another possible inhibitory route to suppress DRN serotonin release.^46,50^ Further studies are needed to establish the direct inhibitory role of adenosine as our technique, FSCV, is limited to detecting rapid changes in electroactive neurotransmitter release and cannot measure changes in basal levels of serotonin.

These studies establish that adenosine is a transient inhibitory signal in the brain and that for serotonin, its own autoreceptors are more important for mediating the effects of adenosine than adenosine receptors. More work is needed to figure out the exact mechanisms of adenosine regulation of DRN serotonin release and how adenosine may interact with other possible inhibitory pathways. However, this study establishes that adenosine is a transiently important modulator of serotonin. Mechanical stimulation or shear stress, caused by injuries such as traumatic brain injury, causes transient adenosine release and may suppress serotonin release during transient injury events.^4,14^ Thus, adenosine is an important modulator of serotonin.

## Conclusions

In this study, we investigated the modulation of adenosine on DRN serotonin release and tested the effects of adenosine receptors (A_1_, A_2A_, and A_3_) and 5HT_1A_ autoregulatory receptors to mediate these effects in real-time using FSCV. Transient adenosine significantly inhibited serotonergic release in the first 20 s of adenosine injection, when adenosine was elevated, and recovered to baseline as the adenosine was cleared from the extracellular space. There were no changes in stimulated serotonin release during continuous adenosine perfusion. Surprisingly, inhibitory effects were still observed in A_1_ knockout mice in the first 10 s of adenosine injection. Moreover, the perfusion of an A_1_ antagonist or an A_3_ antagonist drug did not abolish adenosine inhibition of serotonin release. Similarly, there were no changes in adenosine modulation of serotonin release in A_2A_ knockout mice with or without an A_1_ antagonist. Thus, adenosine inhibited serotonin release in all cases, blocking adenosine receptors, indicating adenosine receptors do not directly regulate the inhibition of serotonin release by adenosine. However, perfusing the 5HT_1A_ antagonist (S)-WAY 100135 dihydrochloride abolished the inhibitory effect of transient adenosine on serotonin release. Thus, transient adenosine transiently inhibits serotonin release but does not directly act through adenosine receptors but acts indirectly through 5HT_1A_ autoreceptors, which tightly control serotonin release at DRN. Understanding the neuromodulatory role of adenosine on the release of serotonin helps to investigate and develop new therapeutic approaches associated with injuries in the brain and other diseases of neuromodulation.

## Methods

### Chemicals

All chemical reagents were purchased from Sigma-Aldrich. The aCSF buffer was composed of 126 mM NaCl, 2.5 mM KCl, 1.2 mM NaH_2_PO_4_, 2.4 mM CaCl_2_·2H_2_O, 1.2 mM MgCl_2_·6H_2_O, 25 mM NaHCO_3_, 11 mM glucose, and 15 mM tris(hydroxymethyl) aminomethane dissolved in deionized water (Milli-Q Biocel; Millipore, USA). The aCSF was freshly prepared before the experiments and adjusted to pH 7.4 for brain slice experiments. Adenosine and inosine were purchased from Acros Organics (Morris Plains, NJ, USA). Stock solutions (10 mM) were prepared in 0.1 M perchloric acid and diluted to a 1 mM concentration with aCSF.

DPCPX (A_1_ adenosine receptor antagonist) and MRS 1220 (A_3_ adenosine receptor antagonist) were purchased from Tocris Bioscience (Minneapolis, USA). A 10 μM amount of DPCPX and 5 μM of MRS 1220 were freshly prepared by dissolving in 1 mL of dimethyl sulfoxide (DMSO) through sonication and diluted with an aCSF buffer.

### Electrochemistry

CFMEs were fabricated by aspirating a T-650 carbon fiber (7 μm diameter, Cytec Engineering Materials, West Patterson, NJ) into a glass capillary (1.2 mm OD × 0.68 mm ID, 4 in., A-M systems) using a vacuum pump. The glass capillary was pulled via a horizontal electrode puller (model PE-21, Narishige, Tokyo). The exposed carbon fiber was cut approximately 70–100 μm long using a scalpel under a microscope.

A CFME was dipped into isopropanol alcohol for 10 min to remove any impurities from its surface and backfilled with 1 mM KCl solution before use. An adenosine waveform (−0.4 to +1.45 and back, scan rate: 400 V/s and 10 Hz vs Ag/AgCl reference electrode) was applied at the CFME, and electrochemical data were collected using a Chem-Clamp (Dagan, Minneapolis, MN, U.S.A.). A dopamine waveform with a switching potential has previously been used for serotonin detection,^34^ and here, extending the potential to 1.45 V allowed both adenosine and serotonin to be detected simultaneously with minimal fouling. All data were analyzed by using HDCV analysis software (UNC Chemistry, Chapel Hill, NC, USA).

### Brain Slice Experiments

The Animal Care and Use Committee (ACUC) of the University of Virginia approved all animal work. 6–8 week old wild-type C57BL/6 mice (Jackson Lab), A_1_KO mice, and A_2A_KO mice were anesthetized with isoflurane. Following quick decapitation, the brain was removed from the skull and transferred into 0–5 °C oxygenated aCSF (95% O_2_, 5% CO_2_) for recovery. The brain was quickly mounted on the cold slicing stage, and coronal section slices (400 μm) were prepared via a vibratome (Leica VT1000S, Bannockburn, IL, USA). Slices were transferred into a chamber containing oxygenated aCSF (32 °C) and kept for 30–45 min to recover. Once the slice was moved to the recording chamber, an electrical stim electrode was placed in the DRN. The carbon–fiber electrode was inserted approximately 75 μm deep in the dorsal raphe and equilibrated for 10–15 min with a waveform applied. Electrically stimulated serotonin release was measured with or without puffed adenosine (50 pmol) while applying the adenosine waveform at the CFME. After each stimulation, 10 min was used as a recovery time before another stimulation. Throughout the experiment, aCSF was continuously perfused over the brain slice by a perfusion pump (Watson-Marlow 205U, Wilmington, MA, USA) at a rate of 2 mL/min.

### Pharmacology Experiments

Pharmacological experiments were performed in a 10 s time frame as we saw a significant effect of transient adenosine over serotonin release in both WT and genetically A_1_, A_2_, and A_3_ knocked-out mice. An A_1_ antagonist (DPCPX, 10 μM, K_i_ = 3.9 nM) or an A_3_ antagonist (MRS 1220, 5 μM, K_i_ > 1 μM) was perfused continuously for 30 min to study the role of A_1_ and A_3_ receptors, respectively.^51,52^ Both antagonists were dissolved in 1 mL of DMSO and diluted with aCSF. Electrically stimulated serotonin and predrug scans with adenosine (10 s after exogenously puffed adenosine) were collected perfusing aCSF. Next, the drug was perfused for 30 min. During drug perfusion, serotonin was stimulated after adenosine application and recorded every 10 min. Finally, postdrug-stimulated serotonin (with adenosine) was recorded for recovery. An A_1_ antagonist was used in WT and A_2A_KO mice, whereas an A_3_ antagonist was used in A_1_KO mice. Genetically altered A_1_ receptor knockout and A_2A_ receptor knockout mice were made by the Mustafa lab (Dr. S. Jamal Mustafa, West Virginia University).^53,54^

A selective 5HT_1A_ antagonist [(S)-WAY 100135 dihydrochloride, 5 μM] was perfused to examine the role of the 5HT_1A_ autoreceptor on DRN serotonergic neurons by adenosine. Initially, evoked serotonin was recorded and 5HT_1A_ perfused for 30 min. During this perfusion, serotonin was stimulated at the 20th minute without adenosine and at the 30th minute with adenosine 10 s before. Finally, the perfusion buffer was switched back to aCSF, and stimulation was applied for the recovery measurement. All serotonin releases were normalized and compared with the initial electrically stimulated serotonin release without adenosine injection.

### Statistics

All statistics were performed by GraphPad 9.0 (GraphPad Software Inc., San Diego, USA), and all data are reported as the mean ± standard error of the mean (SEM) for the “*n*” number of mice. A one-way ANOVA test with Dunnett’s post-test was performed to analyze the initial serotonin with the serotonin with puffed adenosine with or without perfused drugs on the brain slice.

## Supporting Information Available

Color plot with adenosine and serotonin, electrically stimulated serotonin measurement after 20 s of adenosine injection at the DRN, and serotonin current in the presence of adenosine in a mouse brain slice (PDF)
