# Supplementary material for: Transient Adenosine Modulates Serotonin Release Indirectly in the Dorsal Raphe Nuclei
Source: ACS Chem Neurosci. 2024 Feb 9;15(4):798–807. doi: 10.1021/acschemneuro.3c00687 (PMC10885004; doi:10.1021/acschemneuro.3c00687)
Supplement: Supplementary file 1 — cn3c00687_si_001.pdf [file cn3c00687_si_001.pdf]

Supplementary Material for:

Transient adenosine modulates serotonin release indirectly in the DRN

Kailash Shrestha, B. Jill Venton*

Department of Chemistry, University of Virginia, VA, 22901, USA

*Corresponding author: jventon@virginia.edu

Supplementary Figure:

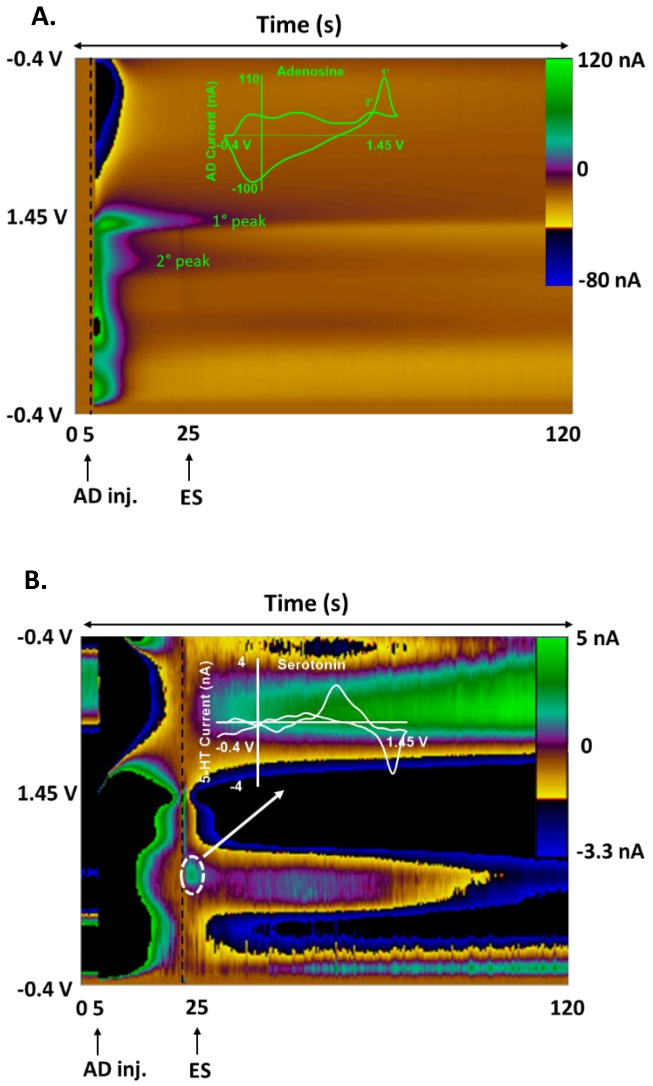

Supplementary Figure 1: Example color plot of adenosine neuromodulation: Electrically stimulated serotonin (at 25 seconds) 20 seconds after adenosine injection (at 5 seconds). The red arrow indicates adenosine (AD) injection and the black arrow indicates electric stimulation (ES). A. Background subtracted color plot and inset adenosine CV, scaled to see adenosine. B. Same color plot, but color scaled to see serotonin; Inset serotonin CV.
